# Auranofin Suppresses Cancer Cell Invasion by Inhibiting Heparanase-1 Expression via the aPKC–NF-κB Pathway

**DOI:** 10.3390/ijms27135646

**Published:** 2026-06-23

**Authors:** Masahiro Komeno, Rin Miyajima, Kanami Miyashita, Masato Suzuki, Toshinao Matoba, Ayuna Miwa, Shoo Katsumoto, Ryosuke Yasumura, Kenta Ko, Hitoshi Kotani, Shoma Tamori, Shoko Itakura, Kosuke Kusamori, Makiya Nishikawa, Kazunori Akimoto, Takashi Suda, Chiaki Takahashi, Nobuaki Higashi, Fuming Zhang, Toshihiko Toida, Kyohei Higashi

**Affiliations:** 1 Faculty of Pharmaceutical Sciences, Tokyo University of Science, 6-3-1 Niijyuku, Katsushika, Tokyo 125-8585, Japan; mkomeno@rs.tus.ac.jp (M.K.); rinelyne1210@icloud.com (R.M.); little520camera@gmail.com (K.M.); masato.suzuki@aist.go.jp (M.S.); toshinao4405@icloud.com (T.M.); kunmthsm.md@gmail.com (A.M.); sho.katsu33_6@yahoo.com (S.K.); 3b26581@ed.tus.ac.jp (R.Y.); tamori@rs.tus.ac.jp (S.T.); itakura@rs.tus.ac.jp (S.I.); kusamori@rs.tus.ac.jp (K.K.); makiya@rs.tus.ac.jp (M.N.); akimoto@rs.tus.ac.jp (K.A.); 2 Graduate School of Pharmaceutical Sciences, Chiba University, 1-8-1 Inohana, Chuo-ku, Chiba 260-8675, Japan; petitsnowdrop5904@gmail.com (K.K.); toida@faculty.chiba-u.jp (T.T.); 3 Faculty of Medicine, Shimane University, Izumo 693-8501, Japan; kotani14@med.shimane-u.ac.jp; 4 Cancer Research Institute, Kanazawa University, Kakuma-machi, Kanazawa 920-1192, Japan; sudat@staff.kanazawa-u.ac.jp (T.S.); chtakaha@staff.kanazawa-u.ac.jp (C.T.); 5 School of Pharmacy and Pharmaceutical Sciences, Hoshi University, 2-4-41, Ebara, Shinagawa-ku, Tokyo 142-8501, Japan; n-higashi@hoshi.ac.jp; 6 Center for Biotechnology and Interdisciplinary Studies, Rensselaer Polytechnic Institute, 110 8th Street, Troy, NY 12180, USA; zhangf2@rpi.edu

**Keywords:** heparanase-1, auranofin, atypical protein kinase C, cancer invasion, hyaluronan, heparan sulfate, epithelial–mesenchymal transition

## Abstract

Heparanase 1 (HPSE1) is the only mammalian endoglycosidase that cleaves heparan sulfate (HS), a glycosaminoglycan (GAG), and is frequently upregulated in cancers, thereby promoting tumor progression. Despite extensive efforts to develop inhibitors of its HS-degrading activity, its non-enzymatic functions limit therapeutic efficacy and pose a major challenge for therapeutic development. Thus, inhibiting HPSE1 expression is critical for controlling its enzymatic and non-enzymatic functions; however, no FDA-approved inhibitors are currently available. Here, we identify auranofin (AUF), an oral gold-containing drug used to treat rheumatoid arthritis, as a potent inhibitor of HPSE1 promoter activity. High-throughput screening revealed that an atypical protein kinase C (aPKC)–NF-κB signaling axis is a key regulator of HPSE1 expression. Notably, AUF treatment reduced HPSE1 expression and significantly suppressed the invasive capacity of MDA-MB-231 cells in a Transwell migration assay. We then investigated the role of HPSE1 in the invasive activity of MDA-MB-231 cells, which produce higher levels of hyaluronan (HA) and HS than non-invasive cells. Neither HS degradation, HA supplementation in Matrigel during Transwell migration, nor HPSE1 overexpression alone was sufficient to drive invasion, suggesting that invasive capacity depends on mesenchymal features and coordinated induction of HPSE1 and GAGs rather than HS degradation. Collectively, our findings demonstrate that AUF-mediated inhibition of aPKC suppresses HPSE1 expression, thereby inhibiting both its enzymatic and non-enzymatic functions and limiting cancer progression, metastasis, and angiogenesis. These results highlight the therapeutic potential of AUF for targeting HPSE1-driven tumor progression and support its repurposing for cancer treatment.

## 1. Introduction

The tumor microenvironment comprises a heterogeneous assemblage of cells, including immune cells, vascular cells, and fibroblasts, embedded within the extracellular matrix (ECM) surrounding tumor cells. Heparanase 1 (HPSE1) is a mammalian endo-β-_D_-glucuronidase playing a critical role in degrading heparan sulfate (HS), a major component of ECM, particularly the basement membrane; it regulates tumor growth, invasion and metastasis, immune evasion, and therapeutic resistance [1,2].

HPSE1 is initially synthesized as a proenzyme (proHPSE1) and localizes at the plasma membrane through interactions with HS on membrane-type proteoglycans [3]. Following endocytosis in complex with HS-containing proteoglycans, the linker region of proHPSE1 is cleaved by cathepsin L, which generates mature HPSE1 within the lysosome. Although the precise mechanism of HS degradation by the mature form of HPSE1 in the ECM remains unclear, partly because of its acidic pH optimum [4], HPSE1-mediated HS degradation and the subsequent release of typical proangiogenic mediators such as platelet-derived growth factor, hepatocyte growth factor (HGF), basic fibroblast growth factor, heparin-binding epithelial growth factor, and vascular endothelial growth factor A (VEGF-A) contributes to the tumor-host crosstalk during ECM remodeling [1,5,6]. Therefore, HPSE1 inhibitors based on polyanionic oligo-/polysaccharides that mimic the physicochemical properties of heparin, a clinically used anticoagulant, have been developed to minimize bleeding risk and to achieve high clinical benefit with minimal adverse effects [6]. However, anticancer therapies targeting HPSE1 are yet to be implemented clinically and remain a major challenge. The adverse effects of major HS mimetic drugs, including poor pharmacokinetics and multifaceted off-target effects, as well as the nonenzymatic functions of HPSE1 contribute to the limitations for the usefulness of enzymatic inhibitors [6]. Indeed, the C-terminal domain (amino acids 413–543) of the extracellularly secreted HPSE1 fragment enhanced protein kinase B (Akt) phosphorylation, facilitating cell proliferation and tumor xenograft progression [7]. Furthermore, HPSE1 also facilitates the phosphorylation of selected signaling molecules including Akt, Src, and epidermal growth factor receptor (EGFR), which in turn enhance the STAT3 phosphorylation correlated with tumor progression [8,9]. Thus, exploring FDA-approved drugs with inhibitory activity against *HPSE1* gene expression may facilitate the development of anti-cancer therapies targeting HPSE1.

Under physiological conditions, HPSE1 expression is largely restricted to endothelial cells, immune cells, and the placenta, partly because of the negative regulation of its promoter activity by DNA methylation [10], histone modifications [11], and p53 [12]. Further, transcription factors such as NF-κB [13], Sp1 [14], ETS [15], and EGR1 [16,17], as well as miRNAs including miR-1258 [18] have been implicated in regulating HPSE1 expression in tumor cells. Although HPSE1 expression is reported to be upregulated by the HIF-1α/IL-1β axis and TNF-α [19], the detailed upstream signaling mechanisms remain largely unknown. Therefore, additional basic research is needed to better understand the role of *HPSE1* gene expression in cancer progression and the associated therapeutic applications.

Here, we found that auranofin (AUF), a thioredoxin reductase inhibitor currently under clinical investigation in combination with chemotherapy, immunotherapy, and targeted therapies [20], markedly suppressed HPSE1 expression and exhibited significant anti-tumor activity. Additionally, the atypical protein kinase C (aPKC)–NF-κB signaling axis plays a critical role in regulating HPSE1 expression in epithelial cancer cell lines.

Notably, invasive cells exhibited higher levels of glycosaminoglycans (GAGs), including hyaluronan (HA) and HS, as well as HPSE1 expression, compared to non-invasive cells. Neither enzymatic degradation of HS in the Matrigel during the Transwell migration assay nor HPSE1 overexpression alone was sufficient to drive invasion, suggesting that invasive capacity depends on mesenchymal features and the coordinated induction of HPSE1 and GAGs rather than solely on HPSE1-mediated HS degradation. Thus, AUF exerts anticancer activity by suppressing HPSE1 expression through inhibition of the aPKC–NF-κB signaling axis, highlighting its therapeutic potential against HPSE1-driven malignancies via both HS degradation-dependent and -independent mechanisms.

## 2. Results

### 2.1. HPSE1 Promoter Activity Was Suppressed by 12 Compounds Capable of Inhibiting PKC or NF-κB in HCT116 Cells

**Figure 1:**
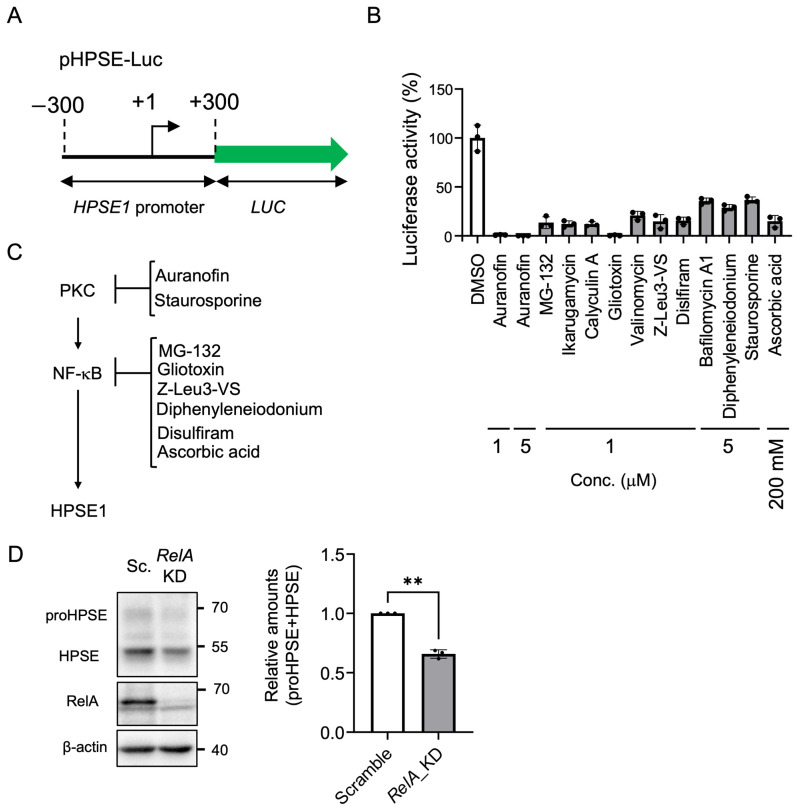
High-throughput screening for inhibitors of HPSE1 promoter activity in HCT116 cells. (**A**) Structure of pHPSE1-Luc. (**B**) Effects of compounds on HPSE1 promoter activity. Luciferase activity was normalized to cell viabilities monitored using the MTT assay. (**C**) The mode of action of these compounds, which inhibit HPSE1 promoter activity, converged on the PKC–NF-κB signaling pathway. (**D**) The effect of *RELA* silencing on the HPSE1 and proHPSE1 protein expression level in HCT116 cells. Twenty micrograms of cell lysate protein was used for western blotting. ** *p* < 0.01; Sc., scramble.

The *HPSE1* promoter region (±300 bp from the transcription start site) including the binding sites of p53, p65 (NF-κB), EGR1, and AP-1 was fused to the luciferase gene to generate the plasmid pHPSE1-Luc for the high-throughput screening for inhibitors of *HPSE1* expression (Figure 1A). HCT116 cells transfected with pHPSE1-Luc were inoculated into a 96-well microplate and further cultured with 1 or 5 μM of 766 compounds from an FDA-approved drug library and 478 compounds from a new ICCB known bioactive library. After 24 h, the cells were mixed with a luciferin solution containing Triton X-100 and luciferase activity was measured at 562 nm. As 249 compounds decreased the luciferase activity by >80% (Table S1), luciferase activities were then normalized to cell viabilities. As shown in Figure 1B, 12 compounds were found to act as inhibitors for HPSE1 promoter activity, and their known modes of action, including side effects [21,22,23,24,25,26], suggested that HPSE1 expression may be regulated by the PKC–NF-κB signaling axis [27,28,29] (Figure 1C). Regulation of HPSE1 expression by NF-κB (p65: *RelA*) in HCT116 cells was then confirmed by Western blotting (Figure 1D).

### 2.2. Auranofin Suppressed HPSE1 Expression by Inhibiting the aPKC-NF-κB Axis

**Figure 2:**
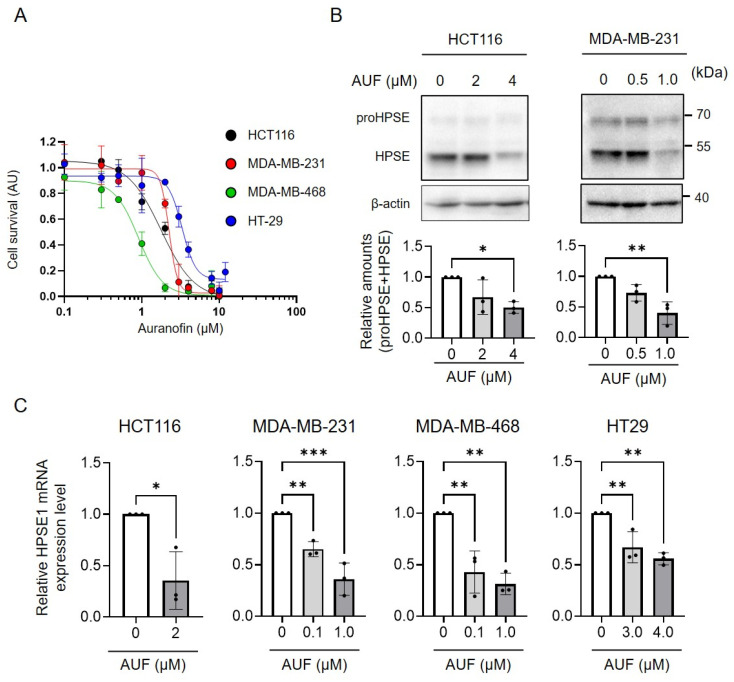
Auranofin suppressed *HPSE1* expression. (**A**) Effect of auranofin (AUF) on cancer cell line survival. The IC_50_ values of AUF for HCT116, MDA-MB-231, MDA-MB-468, and HT-29 cells were 1.77, 2.25, 0.909, and 3.31 μM, respectively. (**B**) AUF reduced the protein levels of HPSE1 and proHPSE1 in HCT116 and MDA-MB-231 cells. Twenty micrograms of cell lysate protein was used for western blotting. (**C**) AUF suppressed HPSE1 mRNA expression in four cancer cell lines. * *p* < 0.05; ** *p* < 0.01; *** *p* < 0.001; ns, not significant.

**Figure 3:**
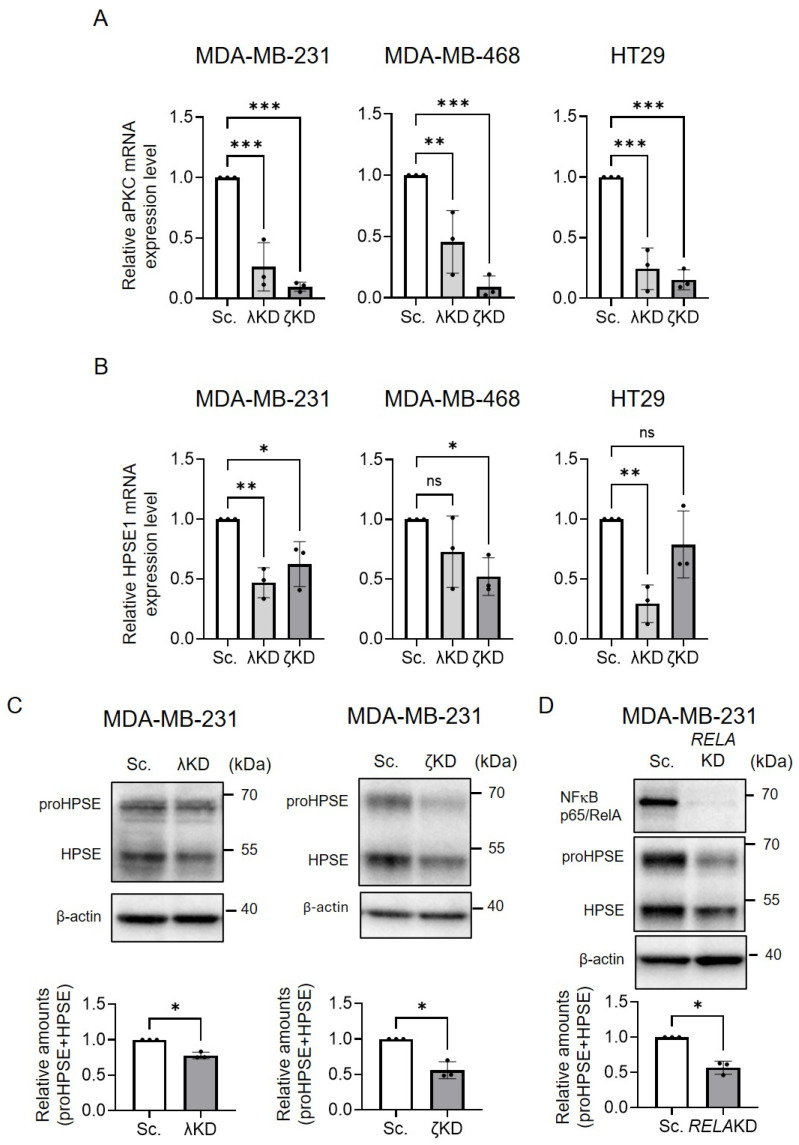
Knockdown of PRKCI or PRKCZ suppresses HPSE1 expression. (**A**,**B**) Decrease in HPSE1 expression in cells transfected with siRNA for *PRKCI* (PKCλ/ι) and *PRKCZ* (PKCζ). (**C**,**D**) Protein levels of HPSE1 and proHPSE1 in MDA-MB-231 cells were suppressed by the silencing of *PRKCI* and *PRKCZ* (**C**) and *RELA* (**D**). Twenty micrograms of cell lysate protein was used for western blotting. * *p* < 0.05; ** *p* < 0.01; *** *p* < 0.001; ns, not significant; Sc., scramble.

Auranofin (AUF), an oral gold-containing drug initially approved by the US Food and Drug Administration for treating rheumatoid arthritis, targets the thioredoxin and glutathione antioxidant systems and is receiving increasing attention for its antitumor activity [30]. Therefore, the effect of AUF on HPSE1 expression in cancer cells was examined further. AUF decreased the viability of HCT116, MDA-MB-231, MDA-MB-468, and HT-29 cells (Figure 2A), and reduced HPSE1 expression at both the protein and mRNA levels (Figure 2B,C). Several reports indicate that AUF also inhibits the signaling pathways of aPKC isotypes PKCλ/ι and PKCζ [23,31], which regulate NF-κB activity [29,32,33]. We found that gene knockdown (KD) of *PRKCI* (PKCλ/ι) or *PRKCZ* (PKCζ) suppressed the expression level of HPSE1 mRNA in MDA-MB-231, MDA-MB-468, and HT29 cells (Figure 3A,B).

The HPSE1 protein level in MDA-MB-231 cells was also suppressed by the knockdown of *PRKCI*, *PRKCZ*, or *RELA* (p65: NF-κB) genes (Figure 3C,D). Considering that HCT116 and HT-29 are colorectal adenocarcinoma cell lines, whereas MDA-MB-231 and MDA-MB-468 are triple-negative breast cancer cell lines, regulation of HPSE1 expression by aPKC–NF-κB signaling may be shared across cancer types, and AUF may have broad effectiveness in suppressing HPSE1 expression.

### 2.3. Auranofin Suppressed the HPSE1-Mediated Invasive Activity of MDA-MB-231 Cells

**Figure 4:**
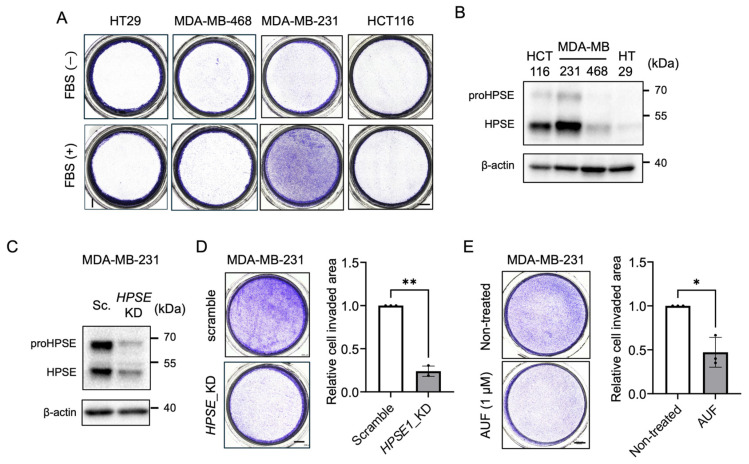
*HPSE1* expression was required for the invasive activity of MDA-MB-231 cells. (**A**) Invasive activity of HT29, MDA-MB-231, MDA-MB-468, and HCT116 cells. (**B**) The expression level of HPSE1 and proHPSE1 proteins in MDA-MB-231 cells was the highest among four cell lines. (**C**–**E**) HPSE1 silencing or AUF treatment reduced the invasive activity of MDA-MB-231 cells. (**C**) Western blotting of HPSE1 protein in MDA-MB-231 cells treated with siRNA targeting HPSE1. (**D**,**E**) Invasive activity of MDA-MB-231 cells treated with siRNA targeting HPSE1 (**D**) and AUF (**E**). Twenty micrograms of cell lysate protein was used for Western blotting. Scale bar, 1 mm. * *p* < 0.05; ** *p* < 0.01.

Myeloma cells expressing high levels of HPSE1 are known to exhibit greater invasive activity than that of cells expressing low levels [34]. Further, the monoclonal antibody (A54) for HPSE1 was found to inhibit the invasion associated with HPSE1-mediated HS-degrading activity in U87 glioma cells [35]. Transwell migration assay with Matrigel containing HS is powerful tool for evaluating the effectiveness of drugs in suppressing cancer cell invasion in vitro [36]. Therefore, correlation between HPSE1 expression levels and invasion activity was investigated using HT-29, HCT116, MDA-MB-468, and MDA-MB-231 cells. Invasive activity in MDA-MB-231 cells was the highest among the four cell lines and positively correlated with HPSE1 protein level (Figure 4A,B). *HPSE1* silencing and AUF reduced the invasiveness of MDA-MB-231 cells (Figure 4C–E). These results suggest that HPSE1 expression is required for the invasive activity of MDA-MB-231 cells and that AUF is an effective agent for suppressing HPSE1-mediated invasive activity.

### 2.4. Auranofin Suppressed HPSE1 Expression in MDA-MB-231 Xenograft-Bearing Mice

Previous reports have suggested that vitamin C (VC: ascorbic acid) potentiates the anticancer effect of AUF in MDA-MB-231 xenografts-bearing mice [30]. As VC was found to inhibit HPSE1 promoter activity (Figure 1B), the effect of AUF with or without VC on the expression level of HPSE1 in tumor tissues of MDA-MB-231 xenografts was examined. As previously reported [30], the body weight of mice administered AUF at 10 mg/kg (AUF), or AUF at 10 mg/kg combined with VC at 1 g/kg (AUF + VC) was almost the same as that of the control mice administered DMSO + PBS (Figure S1A); however, a single dose of AUF reduced tumor volume similar to the AUF + VC combination (Figure S1B). The expression level of HPSE1 in MDA-MB-231 xenografts was reduced by a single dose of AUF or AUF + VC (Figure S1C,D). These results suggest that AUF can suppress HPSE1 expression, which may be regulated by aPKC–NF-κB signaling in vivo.

### 2.5. Coordinated Induction of HPSE1 and GAGs in Cells with a Mesenchymal Phenotype

**Figure 5:**
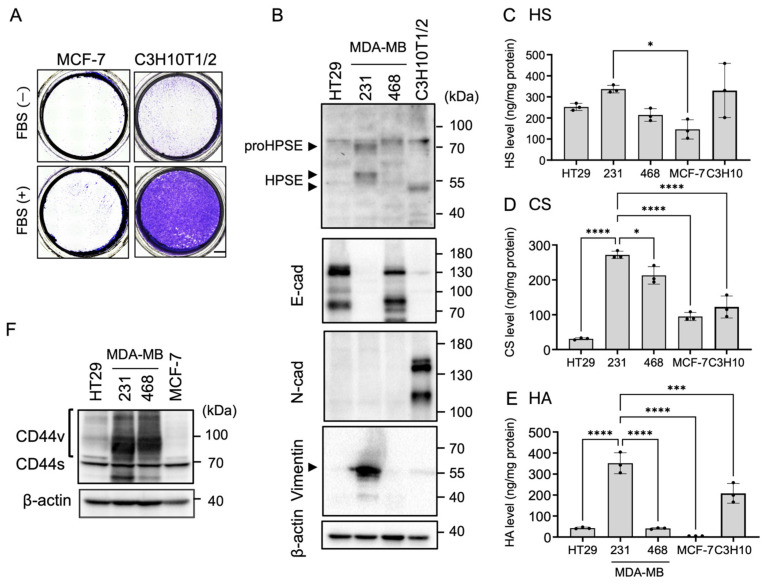
The levels of HPSE1 and HS/HA in invasive cells were higher than those in non-invasive cells. (**A**) Invasive activity of MCF-7 and C3H10T1/2. (**B**) Levels of HPSE1, E-cadherin (E-cad), N-cadherin (N-cad), and vimentin in HT29, MDA-MB-231, MDA-MB-468, and C3H10T1/2 cells. Scale bar, 1 mm. (**C**–**F**) Levels of heparan sulfate (HS) (**C**), chondroitin sulfate (CS) (**D**), hyaluronan (HA) (**E**), and CD44 in cancer cell lines. The levels of HS and CS were expressed as the total amounts of unsaturated disaccharides shown in Figure S2. Twenty micrograms of cell lysate protein was used for Western blotting. * *p* < 0.05; *** *p* < 0.001; **** *p* < 0.0001; ns, not significant.

We investigated whether HPSE1-mediated HS degradation in Matrigel is required for invasive activity, given that HPSE1 is processed into its mature form in lysosomes and exhibits optimal activity at around pH 5. The mouse embryonic mesenchymal stem cell line C3H10T1/2 and the human breast cancer cell line MCF-7 were also included in this study. C3H10T1/2 cells exhibited mesenchymal-like features, similar to those of MDA-MB-231 cells (Figure 5A). In contrast, MCF-7 cells exhibited epithelial features, similar to HCT116, HT29, and MDA-MB-468 cells, based on their morphology and lack of invasive activity. The expression levels of epithelial–mesenchymal transition (EMT)-related proteins including N-cadherin and vimentin were investigated. As previously reported [37,38], MDA-MB-231 cells lack E-cadherin and express vimentin instead of N-cadherin (Figure 5B). C3H10T1/2 cells express N-cadherin, and the HPSE1 expression level in these cells is comparable to that in MDA-MB-231 cells (Figure 5B). Disaccharide analysis suggested that higher levels of HS and HA were characteristic of MDA-MB-231 and C3H10T1/2 cells compared with those of other epithelial cells (Figure 5C–E), indicating that HPSE1-mediated enzymatic activity around the cell body may be quite low. On the other hand, disaccharide compositions of HS in MD-MB-231 and C3H10T1/2 were comparable to those in HT29 cells, whereas the sulfation pattern of CS appeared to be dependent on cell type (Figure S2). Although CD44, a receptor for HA, which promotes invasiveness in MDA-MB-231 cells [39], was highly expressed in both MDA-MB-231 and MDA-MB-468 cells (Figure 5F), MDA-MB-468 cells did not exhibit invasive activity (Figure S3A). Adding HA or CS to Matrigel during coating of the Chemotaxel membrane did not promote invasive activity in MDA-MB-468 cells (Figure S3A). Further, adding chondroitinase AC II to Matrigel for degrading endogenous HA in MDA-MB-231 cells showed no effect on invasive activity (Figure S3B). HPSE1 is known to regulate the expression level of EMT and stemness markers in prostate cancer cell lines [40]. Neither heparinase III-mediated degradation of HS in Matrigel nor transfection with HPSE1 cDNA stimulated their invasive activity (Figure S3C–E). These results suggest that CD44 signaling via interaction with HA, along with HPSE1 expression contribute to invasive activity, but the induction of each factor alone is insufficient to confer invasiveness.

### 2.6. Mesenchymal Properties Are Required for the Invasiveness of Breast Cancer Cell Lines

**Figure 6:**
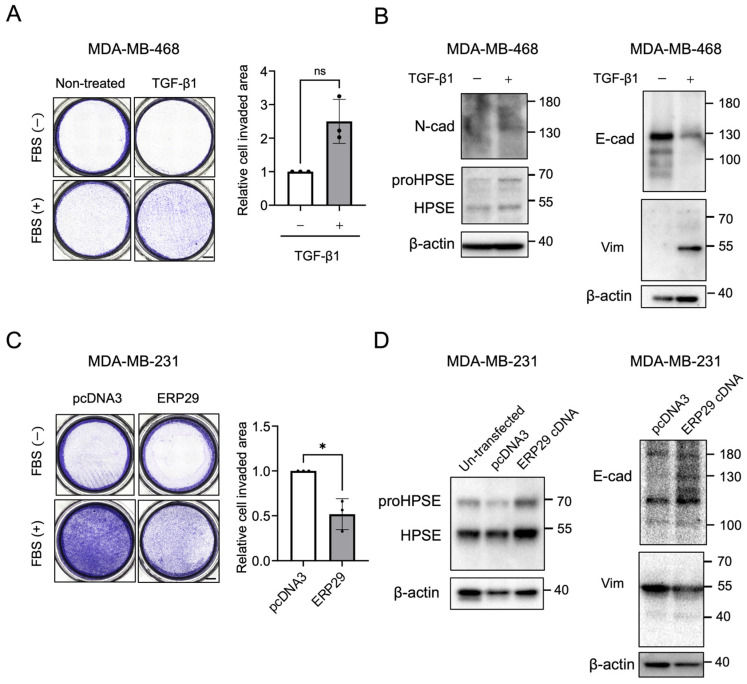
Mesenchymal properties determine the invasive activity of breast cancer cell lines. (**A**,**B**) Effect of TGF-β1 on invasive activity (**A**) and the protein levels of HPSE1 and EMT markers (**B**) in MDA-MB-468 cells. (**C**,**D**) Effect of ERP29 expression on invasive activity (**C**) and the protein levels of HPSE1 and EMT markers (**D**) in MDA-MB-231 cells. Twenty micrograms of cell lysate protein was used for Western blotting. Scale bar, 1 mm. * *p* < 0.05; ns, not significant.

We examined whether epithelial–mesenchymal transition (EMT) stimulated invasion activity when MDA-MB-468 cells were treated with transforming growth factor-beta (TGF-β). In MDA-MB-468 cells treated with TGF-β, N-cadherin and vimentin were upregulated and E-cadherin was downregulated, stimulating moderately invasive activity and *HPSE1* expression compared with that of MDA-MB-231 cells (Figure 6A,B). In contrast, transient ERP29 expression is known to induce mesenchymal–epithelial transition (MET) in MDA-MB-231 cells [41]. Therefore, MDA-MB231 cells were transfected with ERP29 cDNA and cultured for one month in the presence of puromycin. ERP29 transfection of MDA-MB231 cells upregulated E-cadherin and downregulated vimentin, resulting in the repression of invasive activity despite no change in *HPSE1* expression (Figure 6C,D). Overall, these results indicate that mesenchymal properties are required for cell invasiveness.

## 3. Discussion

HPSE1, the only enzyme responsible for HS degradation, causes ECM remodeling and promotes cancer progression, metastasis, and angiogenesis. Clinical trials to date have evaluated heparin/HS mimetics designed to competitively target HS by occupying the HPSE1 binding site; however, none of these mimetics have been approved for clinical use. In addition to the adverse effects of major HS-mimetic drugs, including poor pharmacokinetics and multifaceted off-target effects, the non-enzymatic function of HPSE1 may also contribute, at least partially, to their limited efficacy.

These observations led us to undertake the high-throughput screening of an FDA-approved drug library (Japan Version 2; ENZO, CB-BML-2843J, Version 1.3; 766 compounds), and the results suggested that AUF, disulfiram, and ascorbic acid are potential inhibitors of *HPSE1* expression. Among these, the anticancer effects of AUF, including inhibition of thioredoxin reductase, induction of reactive oxygen species, and modulation of apoptotic pathways, have attracted increasing attention, and AUF has been examined in combination with other cancer treatments, such as chemotherapy, immunotherapy, and targeted therapies, in clinical trials [20]. Further, an ICCB Known Bioactives Library (Japan Version; ENZO, CB-BML-2840J, Version 2.2; 478 compounds) was used to clarify the regulatory mechanisms of *HPSE1* expression. The mode of action of the compounds identified in this study was found to be associated with PKC–NF-κB signaling, whereas involvement of other transcription factors, including Sp1 [14], ETS [15], and EGR1 [16,17], was not observed, suggesting that NF-κB–mediated *HPSE1* expression may also be cell type-specific. Although Sp1 is also regulated by PKCλ/ι [42], regulation of EGR1 and ETS by aPKC isotypes remains unclear. Studies are currently in progress to examine the effects of AUF on endogenous *HPSE1* expression in thyroid cancer cell lines (Sp1 [14]), bladder cancer cell lines (EGR1 [16,17]), and breast cancer cell lines (ETS [15]).

In this study, MDA-MB-231 cells, which exhibit mesenchymal-like properties, showed high levels of HA/HS, CD44, and HPSE1 proteins, along with strong invasive activity. Several reports indicate that *HPSE1* gene expression is related to EMT and cancer stem properties in prostate cancers [40]. CD44 standard (CD44s) and specific CD44 variant (CD44v) isoforms activate various signaling pathways leading to cell proliferation, adhesion, migration, and invasion, via interaction with HA [43,44]. HA accumulation is correlated with poor prognosis in patients with advanced cancers [45,46,47]. In addition to interaction with CD44 and HA, HA and its production, which regulate cancer stem cell-like properties by altering cellular metabolism, have been attracting increasing attention [48,49]. ROR1 functions as a receptor for CS and regulates invasive activity [50]. Considering these findings, we investigated the role of HPSE in conjunction with GAGs and CD44 in the invasive activity of MDA-MB-231 cells. Neither degradation of HA produced by MDA-MB-231 cells using chondroitinase ACII, supplementation with CS/HA, HS degradation by heparinase III in Matrigel, nor transient expression of HPSE1 was sufficient to confer invasive capacity on non-invasive cell lines under our experimental conditions (Figure S3). As the uptake activity of FITC-labeled HA in pancreatic adenocarcinoma cells has been reported [51], we examined HS uptake by MDA-MB-231 and MDA-MB-468 cells. However, FITC-labeled HS uptake was not observed in either cell line. Based on these observations, we concluded that the invasive capacity of MDA-MB-231 cells depends on mesenchymal features and the coordinated induction of HPSE1 and GAGs, rather than on HPSE1-mediated HS degradation. Our results are consistent with previous findings showing that the expression of enzymatically inactive HPSE1 promotes invasion in mouse Eb (L5178Y) T lymphoma cells [52]. As the invasive activity of myeloma cells and U87 glioma cells require HPSE1-mediated HS-degrading activity [34,35], we considered that the requirement for HPSE1-mediated HS-degrading activity in invasion may be cell type-specific. Thus, AUF and aPKC inhibitors may be useful for suppressing the HS-degrading and non-enzymatic activities of HPSE1 via downregulation of *HPSE1* gene expression. Further studies are required to confirm the efficacy of AUF in suppressing *HPSE1* expression in additional cell lines and animal models.

In conclusion, high-throughput screening of drug libraries based on *HPSE1* promoter activity suggested that compounds acting via PKC-NF-κB signaling suppress *HPSE1* expression in HCT116 cells. AUF suppressed *HPSE1* expression in vitro and in vivo, respectively. An advantage of aPKC inhibition is that it can suppress both the enzymatic and non-enzymatic activities of HPSE1 across a broad range of cell types, thereby potentially inhibiting cancer progression, metastasis, and angiogenesis through regulation of gene expression.

## 4. Materials and Methods

### 4.1. Materials

The FDA approved drug library Japan version2, ENZO; CB-BML-2843J, Version 1.3 (766 drugs) and ICCB known bioactives library Japan version, ENZO; CB-BML-2840J Version 2.2 (478 drugs) were obtained from Enzo Life Sciences Inc. (Farmingdale, NY, USA). Auranofin, disulfiram, and ascorbic acid were obtained FUJIFILM WAKO. All other chemicals were of analytical grade. Actinase E (*Streptomyces griseus*) was purchased from Kaken Pharma Co., Ltd. (Tokyo, Japan). Recombinant chondroitinase ABC (*Proteus vulgaris*), chondroitinase ACII (*Arthrobacter aurescens*), heparinase I (*Flavobacterium heparinum*), heparinase II (*Flavobacterium heparinum*), and heparinase III (*Flavobacterium heparinum*) were expressed in *E. coli* BL21 (DE3) and purified as previously described [53]. CS unsaturated disaccharides (ΔDi-0S_CS_, ΔDi-4S, ΔDi-6S_CS_, ΔDiUA-2S, ΔDi-diS_E_, ΔDi-diS_B_, ΔDi-diS_D_, ΔDi-TriS_CS_), HS unsaturated disaccharides (ΔDi-0S_HS_, ΔDi-NS, ΔDi-6S_HS_, ΔDi-diNS6S, ΔDi-diUA2SNS, ΔDi-TriS_HS_), ΔDi-HA were obtained from Seikagaku Corp. (Tokyo, Japan)., New Zealand Pharmaceuticals, Ltd. (Palmerston North, New Zealand), and FUJIFILM Wako Pure Chemical Corporation (Osaka, Japan), respectively. Heparan sulfate (Lot#HO-10697) was obtained from Celsus Laboratories Inc. (Cincinnati, OH, USA).

### 4.2. Cell Culture

HCT116, HEK293, HT-29, MDA-MB-231, MDA-MB-468, MCF7, and C3H10T1/2 cells were purchased from the American Type Culture Collection (ATCC) (Manassas, VA, USA). All cells were cultured in Dulbecco’s Modified Eagle Medium (DMEM; 4.5 g/L glucose) (Nacalai Tesque Inc., Kyoto, Japan) supplemented with 10% FBS (Thermo Fisher Scientific Inc., Bartlesville, OK, USA), 100 units/mL penicillin G and 50 units/mL streptomycin (Nacalai Tesque Inc., Kyoto, Japan) in an atmosphere of 5% CO_2_/95% air at 37 °C. To examine the effect of AUF on HPSE expression and invasion activity, HCT116 (2.0 × 10^4^ cells/mL), HT-29 (4.0 × 10^4^ cells/mL), MDA-MB-231 (4.0 × 10^4^ cells/mL), and MDA-MB-468 (5.0 × 10^4^ cells/mL) were cultured for 6 days in the presence of AUF at the specified concentrations. In the absence of AUF, a two-fold smaller number of cells was cultured. A fusion plasmid in which pcDNA3 was combined with a cDNA encoding human *ERP29* (NM_006817.4) was purchased from VectorBuilder Inc. (Chicago, IL, USA). pcDNA3 and pcDNA-hERP29 were transfected using Multifectam (Promega Co., Madison, WI, USA) with minor modifications to the manufacturer’s instructions. Briefly, 1.5 µL of plasmid (1 µg/mL) was mixed with 1.5 µL of 40 mM Tris-HCl (pH 7.4), followed by adding 46 µL of Multifectam; the mixture was incubated at room temperature for 30 min. Then, 150 µL of Opti-MEM^®^ I (Thermo Fisher Scientific Inc., Bartlesville, OK, USA) was added to the plasmid–Multifectam mixture, which was then mixed with 2 mL of DMEM containing suspended MDA-MB-231 (2.0 × 10^5^ cells) and seeded into culture dishes. After 24 h of culture, the medium was replaced with fresh medium, and the cells were subsequently cultured for approximately 1 month with repeated passaging every 3 days in the presence of 1.5 µg/mL puromycin to establish hERP29-overexpressing MDA-MB-231 cells. As a control, MDA-MB-231 cells transfected with pcDNA3 were cultured for 1 month with repeated passaging every 3 days in the presence of 1 mg/mL neomycin.

### 4.3. Effect of Drugs on Cell Survival

Cell survival rates were measured using an MTT Cell Count kit (Nacalai Tesque Inc., Kyoto, Japan). HCT116 (4.5 × 10^3^ cells), HT-29 (1.8 × 10^4^ cells), MDA-MB-231 (1.8 × 10^4^ cells), and MDA-MB-468 (1.8 × 10^4^ cells) in 0.1 mL of DMEM + 10% FBS/well were inoculated in a 96-well plate and cultured overnight. After culturing for 24 h, 10 μL of drug solutions at the specified concentrations were added and cultured further. After 72 h, the culture medium was changed to 0.1 mL of DMEM without 10% FBS and 10 μL of MTT solution was added. After 1–3 h, 0.1 mL of solubilization solution was added, mixed well, and incubated overnight at 37 °C. An Envision TM 2104 Multilabel Reader (PerkinElmer Inc., Waltham, MA, USA) was used to measure the absorbance at 570 nm.

### 4.4. Screening of HPSE Expression Inhibitors from the Drug Library

Genomic DNA from HEK293 cells was extracted using the DNeasy Tissue kit (Qiagen GmbH, Hilden, Germany). PCR was performed using 5′-CGCGGTACCTGGGTGGTTGATCTCTTTCC-3′ and 5′-TATGCTAGCCCGCCGAGCCCCAGCGCCC-3′ as primers, and genomic DNA was used as the template to amplify the *HPSE1* promoter region (600 bp). The amplified *HPSE* gene was digested with KpnI (TOYOBO Co., Ltd., Osaka, Japan) and NheI (New England Biolabs Inc., Ipswich, MA, USA). The digest was inserted into the same restriction site of pGL4.13 (Promega Corporation, Madison, WI, USA) to generate p*HPSE*-Luc. p*HPSE*-Luc was transfected into HCT116 cells as described by Fukumoto et al. [54] with minor modifications. Briefly, 3.2 µg of plasmid was mixed with 16 µg of polyethyleneimine (Polysciences Inc., Warrington, PA, USA) in 100 µL of buffer A (20 mM sodium lactate, 150 mM NaCl [pH 4.0]) and allowed to stand for 20 min at room temperature. Subsequently, 500 µL of Opti-MEM^®^I (Thermo Fisher Scientific Inc., Bartlesville, OK, USA) was added. Next, 2.4 × 10^5^ cells/well in 6-well plates were cultured for 24 h. After changing the medium to fresh medium containing FBS, the cells were transfected with 600 µL of the plasmid/polyethyleneimine complex in Opti-MEM^®^I and cultured in DMEM containing FBS for 8 h. After replacing the culture medium with fresh medium, the cells were cultured for 24 h. The transfected cells were then collected following trypsin treatment (0.25 *w*/*v*% Trypsin-1 mmol/L EDTA 4Na solution) (Nacalai Tesque Inc., Kyoto, Japan). The transfected cells in suspension (3.0 × 10^3^ cells in 50 μL/well) were inoculated into a CELLSTAR 96 well microplate (Greiner Bio-One, Frickenhausen, Germany) and further cultured for 24 h in the presence of 1 or 5 μM of drugs from the FDA approved library Japan version (766 drugs) and ICCB known Bioactive Library (478 drugs).

Luciferase activity was measured at 562 nm using the Envision^TM^ 2104 Multilabel Reader (PerkinElmer Inc., Waltham, MA, USA) as described previously [36]. To evaluate the cell toxicity of drugs used in this study, an MTT assay was performed as mentioned above. HCT116 cells were inoculated into a 96-well microplate (1.8 × 10^4^ cells in 100 μL/well) and cultured overnight.

### 4.5. Western Blot Analysis

Western blotting was performed as described by Nielsen et al. [55], using Chemi-Lumi One Ultra (Nacalai Tesque, Tokyo, Japan). The antibodies used in this study are listed in Table S2. Antibodies for HPSE (ab254254) and β-actin (mAbcam8226) were obtained from Abcam Limited (Cambridge, UK). RELA/NF-κB p65 Antibody (sc-8008) was purchased from Santa Cruz Biotechnology (Dallas, TX, USA). N-cadherin (22018-1-AP), E-cadherin (20874-1-AP), and vimentin (10366-1-AP) were purchased from Proteintech (Rosemont, IL, USA). CD44 (GTX102111) was purchased from Gene Tex (Irvine, CA, USA). Each antibody was diluted with Signal Enhancer HIKARI (Nacalai Tesque, Tokyo, Japan) or 1 × TBS-T buffer (10 mM Tris-HCl, 150 mM NaCl, 0.05% Tween20). Protein levels were quantified using the ChemiDoc^TM^ MP Imaging System (Bio-Rad Laboratories Inc., Hercules, CA, USA). Protein content was determined using the method described by Lowry et al. [56]. The experiment was performed using lysates obtained from three independent culture dishes. Band densities were analyzed using ImageJ software (Ver. 1.54t; National Institutes of Health, Bethesda, MD, USA).

### 4.6. Measurement of mRNA

Total RNA was isolated using the RNeasy Mini Kit (Qiagen GmbH, Hilden, Germany) according to the manufacturer’s instructions. cDNA was synthesized from 1 µg of RNA using Prime Script RT Master Mix (TaKaRa Bio Inc., Shiga, Japan).

The PrimeTime Std qPCR Assay (Hs.PT.58.15529804) including the probe and primers for measuring *HPSE1* mRNA was obtained from Integrated DNA Technologies Inc. (Coralville, IA, USA). The probes and primers for *PRKCZ* (PKCζ) and *PRKCI* (PKCλ) were also purchased from Merck KGaA (Darmstadt, Germany). Eukaryotic 18S rRNA Endogenous control (VICTM/MGB probe primer limited: 4319413E) was from Thermo Fisher Scientific Inc. (Bartlesville, OK, USA). Quantitative PCR (qPCR) with the THUNDERBIRD^TM^ Probe qPCR mix (Toyobo Co., Ltd., Osaka, Japan) was performed to measure the transcript levels of *HPSE1*, *PRKCZ* (PKCζ), *PRKCI* (PKCλ), and 18S rRNA. The disclosed sequences of primers and probes used for mRNA measurements are listed in Table S3.

### 4.7. Gene Knockdown

The siRNA for the *HPSE1* gene was from Sigma-Aldrich Co. (Saint Louis, MO, USA). DsiRNAs for the *PRKCI* gene and negative control were obtained from Integrated DNA Technologies Inc. (Coralville, IA, USA). The expression of PKCλ was silenced using DsiRNA #1 in MDA-MB-231 cells, whereas DsiRNA #2 was used in HT29 and MDA-MB-468 cells (Table S3). siRNAs for *PRKCZ* and *RELA* genes were purchased from Merck Millipore, Ltd. (Darmstadt, Germany). Silencer Select Control No.1 siRNA (catalogue no. 4390843) used as the scrambled control was from Thermo Fisher Scientific Inc. (Bartlesville, OK, USA).

Transfection of siRNA was performed using Lipofectamine^®^ RNAiMAX (Thermo Fisher Scientific Inc., Bartlesville, OK, USA) according to the manufacturer’s instructions. Briefly, 30 pmol of siRNA was mixed with 5 µL of Lipofectamine^®^ RNAiMAX in 500 µL of Opti-MEM and allowed to stand for 20 min at room temperature. HT-29 (1.5 × 10^5^ cells) and MDA-MB-231 (1.5 × 10^5^ cells) in 2 mL DMEM with 10% FBS were combined with 500 µL of siRNA/Lipofectamine^®^ RNAiMAX complex in 6-well plates and cultured for 24 h. For MDA-MB-468, 1.5 × 10^5^ cells in 2 mL DMEM with 10% FBS were cultured for 24 h followed by adding 500 µL of the siRNA/Lipofectamine^®^ RNAiMAX complex to the culture medium and further culturing for 24 h. After replacing the medium with fresh medium, the cells were again transfected with 500 µL of siRNA/Lipofectamine^®^ RNAiMAX complex and cultured in DMEM with 10% FBS. After culturing for 48 h, the transfected cells were collected.

### 4.8. Plasmid Transfection of Plasmids

pCMV-h*HPSE1* was obtained from OriGene Technologies Inc. (Rockville, MD, USA). Plasmid transfection into HT-29 and MDA-MB-468 cells was performed as described by Fukumoto et al. [54] with minor modifications. Briefly, 1 µg of plasmid was mixed with 6 µg of polyethyleneimine (Polysciences Inc., Warrington, PA, USA) in 100 µL of buffer (20 mM sodium lactate, 150 mM NaCl [pH 4.0]) and allowed to stand for 20 min at room temperature. Subsequently, 500 µL of Opti-MEM^®^I (Thermo Fisher Scientific Inc., Bartlesville, OK, USA) was added. Next, 1.6 × 10^5^ cells in 6-well plates were cultured for 24 h. After changing the medium to a fresh one containing FBS, the cells were transfected with 600 µL of plasmid/polyethyleneimine complex in Opti-MEM^®^I and cultured in DMEM containing FBS for 8 h. After replacing the culture medium with fresh medium, the cells were cultured for 48 h.

### 4.9. Invasion Assay

To examine the effect of AUF on invasion activity, MDA-MB-231 cells (2.0 × 10^4^ cells/mL) were cultured for 5 days in the presence of 1 µM AUF. To examine the effect of TGF-β1 on invasion activity, MDA-MB-468 cells (2.0 × 10^5^ cells/mL) were cultured for 2 days in the presence of 10 ng/mL TGF-β1. Knockdown of HPSE and overexpression of HPSE or ERP29 were performed as described above. Matrigel Basement Membrane Matrix Growth Factor Reduced (catalog no. 356230, Corning Inc., Corning, NY, USA) diluted to 0.25 mg/mL in serum free DMEM was added to the top membrane surface of an 8 µm pore size of Chemotaxel (Kurabo Industries, Ltd., Osaka, Japan) and incubated for 2 h at 37 °C and 5% CO_2_ conditions. To degrade HS or CS produced by invading cells and those present in Matrigel, 0.5 µg of heparinase III or 0.57 µg of chondroitinase AC II was added. After coating, the Chemotaxel was transferred to a 24-well plate containing 600 µL of DMEM supplemented with 10% FBS. HCT116 cells (5.0 × 10^5^ cells), MDA-MB-231 cells (5.0 × 10^5^ cells), MDA-MB-468 (5.0 × 10^5^ cells), HT-29 (5.0 × 10^5^ cells), and C3H10T1/2 (4.0 × 10^5^ cells) were seeded in 200 µL of DMEM onto the Chemotaxel upper layer. To examine the effects of CS and HA on invasion activity, CS-A purchased from Shin Nippon Yakugyo Co., Ltd. (Tokyo, Japan) and sodium hyaluronan purchased from Kikkoman Biochemifa Co. (Tokyo, Japan) were each added to the Chemotaxel upper layer at a final concentration of 50 µg/mL. After 24 h of culture at 37 °C with 5% CO_2_, cells infiltrating beneath the Chemotaxel membrane were fixed for 15 min in 4% paraformaldehyde-phosphate-buffered saline (PBS) solution. The residual cells and medium on the membrane surface were removed using a cotton swab. Cells infiltrating beneath the membrane were stained for 30 min with a 0.2% Crystal Violet Water Solution (catalog no. 15192; Muto Pure Chemicals Co. Ltd., Tokyo, Japan). After washing with water and removing the residual staining solution from the membrane surface with a cotton swab, the samples were observed using a BZ-X800 microscope (Keyence Corp., Osaka, Japan). The summarized graph data are presented as the mean ± standard deviation obtained from three independent experiments. Images were analyzed using ImageJ software.

### 4.10. Tumor-Bearing Model Mice

All animal experiments were approved by the Institutional Animal Care and Use Committee of Tokyo University of Science and carried out according to the Guidelines for Animal Research of Tokyo University of Science. Female BALB/c-nu/nu mice (5-week-old) were purchased from Japan SLC Inc. (Shizuoka, Japan). Body weight was measured for each mouse. Mice were subsequently allocated to groups of three animals each, with groups balanced for mean body weight. The sample sizes were selected based on previously published results [30]. 6-week-old mice weighing 14 to 19 g were anesthetized by inhalation of 3% isoflurane (Viatris, Tokyo, Japan). MDA-MB-231 cells were suspended in PBS and mixed with MatriMix for PDX (Nippi Inc., Tokyo, Japan) at a 1:1 ratio. The mixture corresponding to 1.0 × 10^6^ cells was subcutaneously injected into the left flank of mice. When the tumor volume reached 20–40 mm^3^, the mice were randomly divided into three groups. AUF was dissolved in DMSO to a final concentration of 5 mM. VC was dissolved in PBS to 755 mM, and pH was adjusted to approximately 7 using NaOH. Mice received intraperitoneal injections of 28% DMSO in PBS (control), AUF at 10 mg/kg (AUF), or AUF at 10 mg/kg combined with VC at 1 g/kg (AUF + VC) once every 24 h for a total of 15 days, excluding Saturdays and Sundays. Tumor size was measured using a ruler, and tumor volume was calculated as $\frac{4}{3}\pi r^{3}$, with the diameter defined as the average of tumor length and width and *r* = diameter/2. After treatment completion, the mice were euthanized under anesthesia, and the tumors were promptly excised and processed for histochemical analysis. No formal power analysis was performed. No technical replicates were included in the statistical analyses.

### 4.11. Immunohistochemical Staining

Tumors were fixed overnight in a 4% paraformaldehyde-PBS solution. The fixed tumors were embedded in Tissue-Tek^®^ O.T.C. Compound (Sakura Finetek USA Inc., Torrance, CA, USA) and frozen overnight at −20 °C to prepare blocks. Frozen tissue sections were prepared using a LEICA CM3050 S (Leica Biosystems, Nussloch, Germany). The samples were permeabilized with 0.1% polyethylene glycol mono-p-isooctylphenyl ether (Nacalai Tesque Inc., Kyoto, Japan) for 10 min. After washing with PBS, the samples were blocked with 4% bovine serum albumin (BSA) (Nacalai Tesque Inc., Kyoto, Japan) for 30 min. The samples were then incubated overnight at 4 °C with anti-human heparanase 1 antibody (INS-26-2-0000-12) (Insight Biopharmaceuticals, Ltd., Rehovot, Israel) diluted in 4% BSA. Following PBS washes, the samples were incubated with Alexa Fluor^TM^ 488 goat anti-rabbit IgG (H + L) (A11008) (Thermo Fisher Scientific Inc., Bartlesville, OK, USA) for 1 h at room temperature, protected from light. After washing with PBS, the samples were mounted with VECTASHIELD antifade mounting medium (Vector Laboratories Inc., Burlingame, CA, USA) containing DAPI solution (Dojindo Laboratories, Kumamoto, Japan). The stained samples were then observed using a BZ-X800 microscope (Keyence Corp., Osaka, Japan). A random field (~67.2 μm^2^ per field) was selected from each of the three sections of the stitched image obtained from each animal. Images were analyzed using ImageJ software.

### 4.12. Disaccharide Analysis of GAGs by High Performance Liquid Chromatography

Glycosaminoglycan extraction from cells was performed with modifications based on the method reported by Yamaguchi et al. [57] Analysis of CS, HS, and HA unsaturated disaccharides was performed as reported by Ko et al. [58].

### 4.13. Statistical Analysis

Values are expressed as means ± SD. Differences between the two groups were analyzed using Student’s *t*-test. One-way analysis of variance (ANOVA), followed by Dunnett’s test, was used to evaluate differences among GAGs and cancer cell lines, as well as among treatment groups in the xenograft study. Data distribution was assumed to be normal, and statistical analyses were conducted using parametric tests. All statistical analyses were performed using GraphPad Prism version 10.4.2 (GraphPad Software, San Diego, CA, USA).

## Data Availability

The original contributions presented in this study are included in the article. Further inquiries can be directed to the corresponding author.

## Supplementary Materials

The following supporting information can be downloaded at https://www.mdpi.com/article/10.3390/ijms27135646/s1.

## Abbreviations

The following abbreviations are used in this manuscript:

aPKC	Atypical protein kinase C
AP-1	Activator protein 1
ATP	Adenosine triphosphate
AUF	Auranofin
BSA	Bovine serum albumin
CS	Chondroitin sulfate
DsiRNA	Dicer-substrate small interfering ribonucleic acid
EGR1	Early growth response protein 1
ER	Estrogen receptor
GAG	Glycosaminoglycan
HA	Hyaluronan
HIF-1	Hypoxia-inducible transcription factor 1
HPSE	Heparanase
HS	Heparan sulfate
Luc	Luciferase
MTT	3-(4,5-Dimethyl-2-thiazolyl)-2,5-diphenyltetrazolium Bromide
NF-κB	Nuclear factor-kappa B
siRNA	Small interfering ribonucleic acid, Sp1: Specificity protein 1.
