# Supplementary material for: Auranofin Suppresses Cancer Cell Invasion by Inhibiting Heparanase-1 Expression via the aPKC–NF-κB Pathway
Source: Int J Mol Sci. 2026 Jun 23;27(13):5646. doi: 10.3390/ijms27135646 (PMC13361064; doi:10.3390/ijms27135646)
Supplement: Supplementary file 1 [file ijms-27-05646-s001.zip › Suuplementary figure_AUF_260618_submitted.pdf]

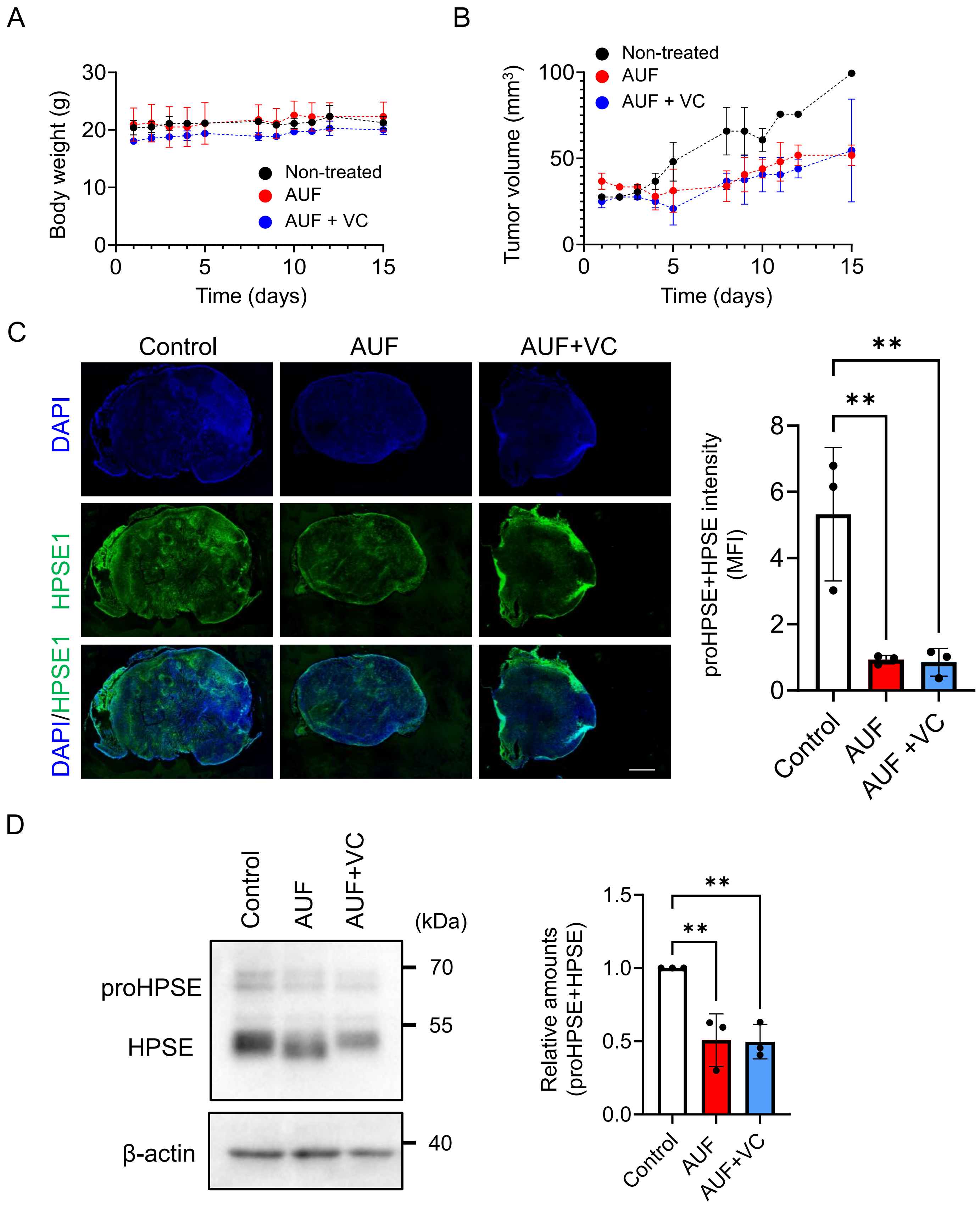
A
Body weight (g)
Time (days)
Non-treated
AUF
AUF + VC
B
Tumor volume (mm³)
Time (days)
Non-treated
AUF
AUF + VC
C
Control
AUF
AUF+VC
DAPI
HPSE1
DAPI/HPSE1
proHPSE+HPSE intensity (MFI)
Control
AUF
AUF +VC
D
Control
AUF
AUF+VC
(kDa)
proHPSE
HPSE
β-actin
70
55
40
Relative amounts (proHPSE+HPSE)
Control
AUF
AUF+VC

Figure S1. Auranofin reduces HPSE1 protein levels in a xenograft mouse model.

(A, B) Effect of auranofin (AUF) on body weight (A) and tumor volume (B). (C, D) Effects of intraperitoneal AUF administration on HPSE1 expression levels in the tumors, evaluated by immunohistochemistry (C) and western blotting (D). Twenty micrograms of tissue lysate proteins was used for western blotting. Mean fluorescence intensity (MFI) was calculated using ImageJ software by dividing the integrated fluorescence intensity by the selected area. Scale bar, 1 mm.

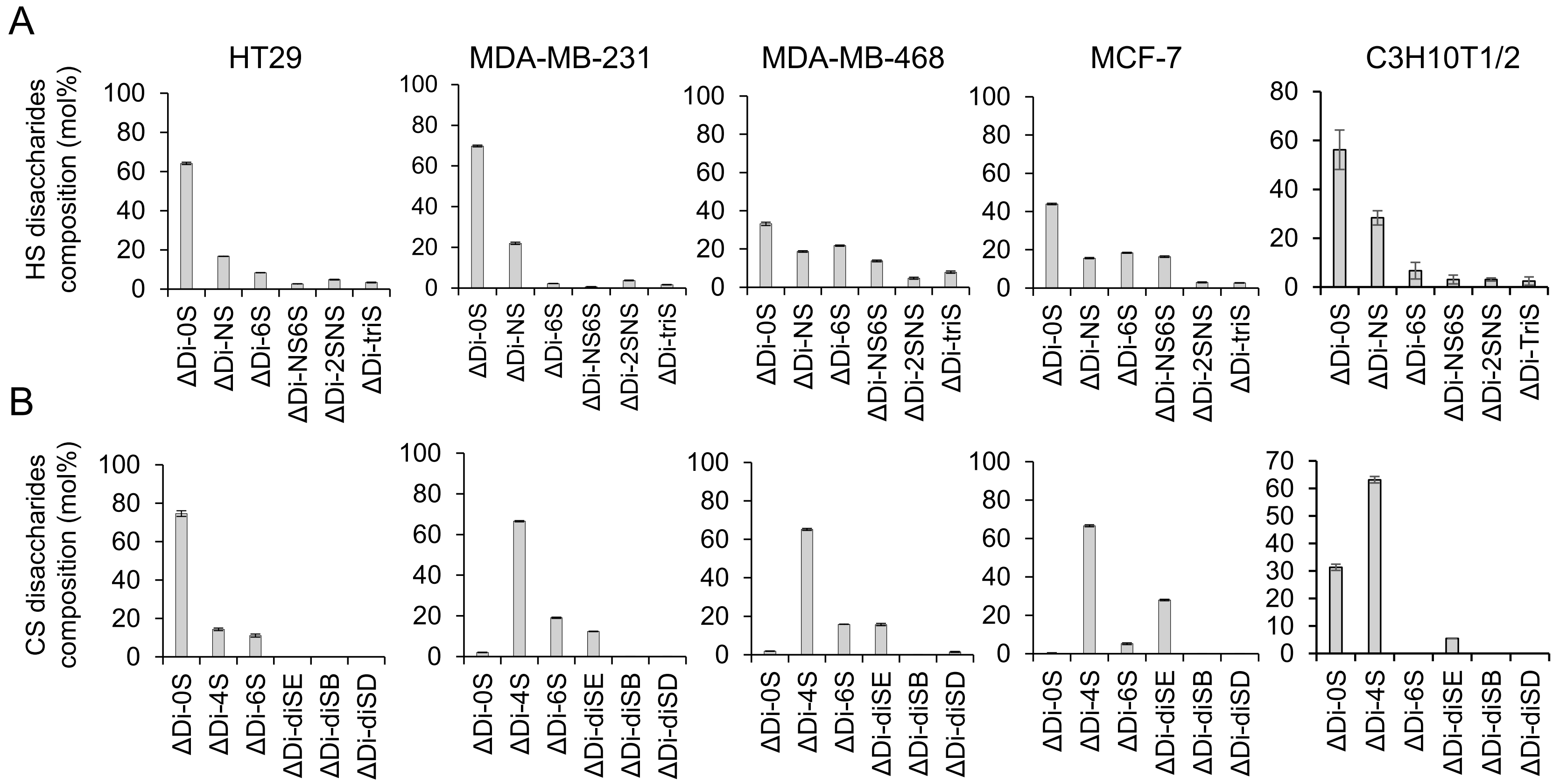

Figure S2. Disaccharide composition of heparan sulfate (A) and chondroitin sulfate (B) in invasive and non-invasive cells.

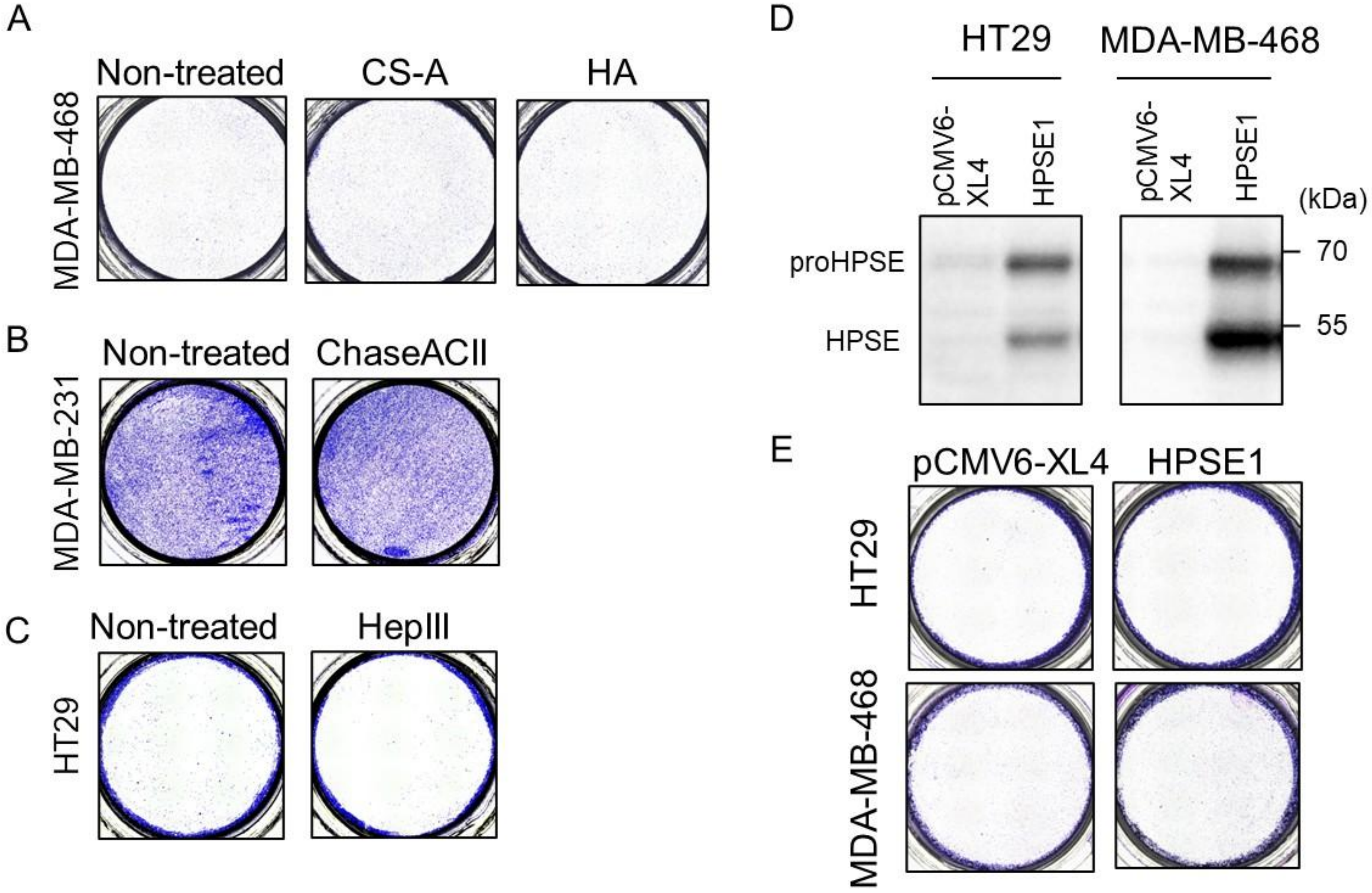

Figure S3. Effect of GAG supplementation in Matrigel (A), degradation of HA produced by MDA-MB-231 cells using chondroitinase ACII (B), degradation of HS in Matrigel by heparinase III (C), and transient *HPSE1* expression (D) on invasive activity.

Preparation of Matrigel containing chondroitinase (Chase) ACII or heparinase (Hep)III, and transfection of HPSE1 cDNA are described under "Materials and Methods"
